# 24-Month results of the BRAVO study: A prospective, multi-center study evaluating the clinical outcomes of a ventral hernia cohort treated with OviTex® 1S permanent reinforced tissue matrix

**DOI:** 10.1016/j.amsu.2022.104745

**Published:** 2022-09-27

**Authors:** George DeNoto, Eugene P. Ceppa, Salvatore J. Pacella, Michael Sawyer, Geoffrey Slayden, Mark Takata, Gary Tuma, Jonathan Yunis

**Affiliations:** a Department of General Surgery, St. Francis Hospital, Roslyn, NY, 11576, USA; b Department of Surgery, Indiana University School of Medicine, Indianapolis, IN, 46202, USA; c Scripps Clinic and Scripps M.D. Anderson Cancer Center, Division of Plastic and Reconstructive Surgery, San Diego, CA, 92130, USA; d Department of Surgery, Comanche County Memorial Hospital, Lawton, OK, 73505, USA; e St. Luke's Surgical Specialists, St. Luke's Health System, Overland Park, KS, 66213, USA; f Scripps Clinic Medical Group, Department of Surgery, La Jolla, CA, 92037, USA; g Capital Health Medical Group, Department of Plastic Surgery, Pennington, NJ, 08534, USA; h Hernia Specialist, Center for Hernia Repair, Sarasota, FL, 34239, USA

**Keywords:** Ventral hernia repair, Reinforced biologic, Ovine reinforced tissue matrix, Hernia recurrence

## Abstract

**Background:**

This study evaluated the performance of OviTex® 1S (TELA Bio Inc., Malvern, PA, USA) over 24 months when used for ventral hernia repair.

**Methods:**

This was a prospective, single-arm, multi-center clinical trial (ClinicalTrials.gov/NCT03074474). A ninety-two patient cohort with ventral hernias were enrolled. The surgical approach (open, laparoscopic, or robotic) and plane of placement (retrorectus, intraperitoneal, or pre-peritoneal) were at the discretion of the surgeon. Patients were characterized as high risk for a surgical site occurrence (SSO) based on the following comorbidities: BMI between 30 and 40, active smoker, chronic obstructive pulmonary disease (COPD), diabetes mellitus, coronary artery disease, advanced age ( ≥ 75 years). Subjects underwent physical examinations to evaluate safety events and completed quality of life surveys at 1 months, 3 months, 12 months, and 24 months post-surgery.

**Results:**

Sixty-five of the 92 enrolled patients (70.7%) completed 24-month follow-up. The Kaplan Meier estimate for risk of recurrence at day 730 (24 months) was 2.6%; among subjects who completed their 24-month visit or had a previous recurrence, the unadjusted rate of recurrence was 4.5% (3/66). SSOs were observed in 38.0% of patients (35/92). The most prevalent SSO was surgical site infection occurring in 20.7% (19/92) of patients, followed by seroma formation, which occurred in 13.0% of patients; however, only 3.3% required intervention. HerQLes and EQ-5D assessments showed improvement from baseline as soon as 3 months post-surgery. Continued improvement was observed through 24 months

**Conclusions:**

Overall the BRAVO study demonstrates that use of the ovine reinforced tissue matrix OviTex 1S is a viable option for use in ventral hernia repair. Additional studies with longer term follow-up data are needed to draw definitive conclusions on the use of OviTex 1S.

## Highlights

• Prospective, multi-center, single arm study of OviTex 1S reinforced biologic material for ventral hernia repair.
• 24-month results show low hernia recurrence rate and acceptable SSO rate.
• Statistically significant and clinically meaningful improvement in patient self-reported quality of life over 24 months.

## 1 Introduction

Ventral hernias occur in 1.7% of the US population, and the prevalence increases to 4% in patients over 45 years of age [1,2]. Ventral hernia repair techniques aim to reduce complications and recurrence rates, which are reported between 5.6 and 40% [[2], [3]] The first prosthetics to reinforce hernia repairs were synthetic polymer meshes which are still the most common prosthetic on the market today [4]. In addition to traditional permanent synthetic meshes, resorbable synthetic meshes, biologic-based matrices, and reinforced biologic matrices are now also available. Use of any mesh or matrix device has proven effective in reducing hernia recurrence compared to suture technique alone and has become the gold standard recommended by the Ventral Hernia Working Group (VHWG) [[5], [6], [7], [8]]. The use of mesh in ventral hernia repair can be complicated by infection, pain, adhesions, mesh extrusion, and hernia recurrence [9]. Mesh or matrix type may influence these complication rates. There is some evidence that permanent synthetic meshes are more prone to complications like infections, and alternatively that pure biologics may not provide enough long-term strength leading to recurrence [3,[10], [11], [12], [13], [14], [15]]. Reinforced biologics offer an alternative, designed with a biologic base to facilitate tissue remodeling and a low synthetic polymer footprint for reinforcement.

This study evaluated 92 patients who underwent ventral hernia repair with OviTex 1S, an ovine reinforced tissue matrix. OviTex 1S is comprised of 6 layers of decellularized ovine forestomach extracellular matrix (OFM) reinforced with 5% permanent or resorbable polymer embroidery, with permanent polymer reinforcement used for the BRAVO study.

OviTex 1S has been evaluated in a preclinical full thickness defect model in non-human primates compared to seven commercially available mesh products [16]. The results demonstrated OviTex had a limited foreign body response, earlier cellular infiltration and remodeling. This was attributed to the OFM base material which retains the native structure and extracellular matrix components to promote integration into host tissue, as well as the engineered macroscopic architecture, which contains channels and pores to promote fluid exchange and host cell migration [16].

Based on the favorable pre-clinical healing response, it is hypothesized that OviTex may combine the benefits of a biologic matrix in terms of wound healing and lower inflammatory profiles, with the benefits of a synthetic polymer in terms of maintaining the structural integrity necessary for long term support. The results of the BRAVO study appear to support this hypothesis and provide prospectively collected clinical evidence for the ability of OviTex 1S to minimize recurrence rates, limit serious safety events, and improve patient reported quality of life up to 24 months after ventral hernia repair.

## 2 Materials and methods

### 2.1 Study design

This prospective, single-arm, multi-center clinical trial evaluated OviTex 1S in a 92-patient cohort undergoing ventral hernia repair (ClinicalTrials.gov/NCT03074474). This is the first prospective post-market OviTex clinical trial and it was designed to accommodate a variety of surgical techniques and approaches to evaluate the performance of OviTex under real world conditions. Patients were continuously recruited through physician referral at seven sites with written informed consent obtained from March 2017 through September 2019 including: St. Francis Hospital (Rosyln, NY), Indiana University School of Medicine (Indianapolis, IN), Scripps Clinic Medical Group (San Diego, CA), Scripps M.D. Anderson Cancer Center (San Diego, CA), Comanche County Memorial Hospital (Lawton, OK), St. Luke's Health System (Overland Park, KS), Capital Health Medical Group (Pennington, NJ), and the Center for Hernia Repair (Sarasota, FL). Prior to the enrollment of subjects, the protocol was approved by each center's Institutional Review Board (IRB) or a central IRB (WIRB Pr. No.: 20142056) and aligns with the 1964 Helsinki declaration and its later amendments. This study is reported following the STROCCS guidelines [17].

### 2.2 Inclusion criteria

Subjects were 18 years or older with BMI ≤40 kg/m^2^ and ventral hernias requiring repair with an expected OviTex 1S matrix size no larger than 400 cm^2^ (18 × 22 cm, 20 × 20 cm or less). Patients had ventral hernias meeting the Center for Disease Control and Prevention (CDC) wound classification system of Class I (clean), Class II (Clean-Contaminated), or Class III (Contaminated).

### 2.3 Exclusion criteria

Subjects were excluded if they were younger than 18 years, had a BMI above 40 kg/m^2^, or had a ventral hernia expected to require a matrix larger than 400 cm^2^. Patients with CDC Class IV (Dirty-Infected) wounds, who were pregnant, had a life expectancy of <2 years, had a history of drug or alcohol abuse in the last 3 years, and/or were allergic to ovine-derived products were excluded.

### 2.4 Surgical technique

Ventral hernia repair was achieved using standard operative techniques as previously described [18]. The surgical approach (open, laparoscopic, or robotic) and plane of placement (retrorectus, intraperitoneal, or pre-peritoneal) were at the discretion of the surgeon. All subjects were administered preoperative, intraoperative, and postoperative standard of care according to hospital protocol.

### 2.5 Follow up

Endpoints for the 92 subjects were evaluated at 1, 3, 12, and 24 months. Patients were incentivized to return for follow up visits with a nominal IRB approved stipend. At each follow up visit, patients underwent physical examination to assess post-operative adverse events, surgical complications, or hernia recurrence and patient reported outcome data was collected.

### 2.6 Primary endpoints

The primary endpoints were incidence of SSOs or wound related events at the hernia repair site and incidence of other complications <90 days after index surgery. These included seromas, hematomas, wound dehiscence, skin necrosis, fistulae, and infections. Complications such as ileus and bowel obstruction were also recorded.

### 2.7 Secondary endpoints

The secondary endpoints were incidence of hernia recurrence, incidence of post-operative SSOs and wound related events at the hernia repair site occurring at time points >90 days after index surgery, and incidence of other complications occurring >90 days after index surgery. The EuroQol-5 Dimension (EQ-5D) health assessment and the validated 12-question Hernia-Related Quality of Life survey (HerQLes) were used to assess patient reported quality of life (QoL).

### 2.8 Statistical analysis

Summary statistics were used to analyze the data including the number of subjects who completed follow up at each timepoint, mean, median, standard deviation, minimum, and maximum. For EQ-5D, EQ-5D VAS, and HerQLes QoL scores, 95% confidence intervals (CI) were calculated to determine the certainty of mean values and nominal paired t-tests were utilized to determine any mean change in subsequent timepoints from baseline. Paired differences profiles were calculated for EQ-5D, EQ-5D VAS, and HerQLes 24-month data. Risk factor analyses were performed in which selected safety endpoints were subjected to stratified survival analyses. In these risk factor analyses, cumulative event rates for 3 months (day 90), 12 months (day 365), and 24 months (day 730) were determined using Kaplan Meier analyses. This analysis was used to account for loss to follow up and utilizes all available data to produce valid estimates of cumulative event rates. Ninety-five percent (95%) confidence intervals were also calculated for the cumulative event rates. Chi-square and Kruskal-Wallis tests were performed to analyze differences between the 65 subjects who completed 24-month follow up and the 27 subjects that did not complete 24-month follow up to determine any potential differences between these patient populations.

## 3 Results

### 3.1 Baseline demographics and risk factors

**Fig. 1:**
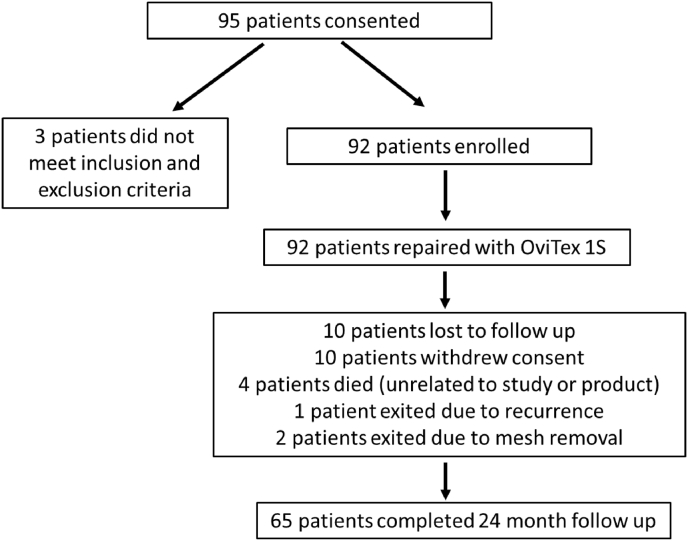
Patient accounting through 24 months.

Sixty-five (65) of the 92 enrolled patients (70.7%) returned for the final study visit at 24 months (Fig. 1). The mean age was 60.42 ± 13.13 years (Table 1). Most patients were female (59.78%), obese (55.43%), and had prior abdominal surgeries (83.70%) (Table 1). Thirty-four (34) patients (36.96%) had prior ventral hernia repairs (Table 1). Most patients (77.17%) presented with complex hernias classified as Grade II (Comorbid) or III (Contaminated) on the modified VHWG scale (Table 1) [19]. Wounds were generally classified as clean (83.08%) on the CDC Wound Classification scale (Table 1). Chi-square and Kruskal-Wallis analyses did not determine any significant differences between subjects with and without 24-month follow up, with exception of age (mean of 63.09 ± 10.75 vs 54.00 ± 16.07 respectively, p = 0.009).

**Table 1:** Baseline demographics and intraoperative variables.

Demographic	Total (N = 92)
**Sex, n (%)**	
Female	55 (59.78%)
Male	37 (40.22%)
**Age**	
N	92
Mean (SD)	60.42 (13.13)
**Body Mass Index (kg/m2)**	
N	92
Mean (SD)	31.00 (4.51)
**BMI between 30 and 40, n (%)**	
No	41 (44.57%)
Yes	51 (55.43%)
**Race, n (%)**	
Black or African American	3 (3.26%)
Asian	1 (1.09%)
White	88(95.65%)
**Ethnicity, n (%)**	
Hispanic	1 (1.09%)
Not Hispanic	91 (98.91%)
**Surgical Site Wound classification, n (%)**	
CDC Class I Clean	74 (80.43%)
CDC Class II Clean-Contaminated	14 (15.22%)
CDC Class III Contaminated	4 (4.35%)
**mVHWG Grade, n (%)**	
Grade I	21 (22.83%)
Grade II	52 (56.52%)
Grade III	19 (20.65%)
**# Prior Surgeries**^a^	
N	88
Mean (SD)	3.89 (3.35)
**Prior Abdominal Surgery, n (%)**	
No	15 (16.30%)
Yes	77 (83.70%)
**Is there any prior ventral hernia repairs to report? n (%)**	
No	58 (63.04%)
Yes	34 (36.96%)
**Total number of prior ventral hernia repairs**	
N	34
Mean (SD)	1.91 (0.97)
**Operative Approach**, **n (%)**	
Open	60 (65.22%)
Laparoscopic	12 (13.04%)
Robotic	20 (21.74%)
**Hernia Defect Size (cm^2^)**	
N	92
Mean (SD)	112.05 (127.73)
**Hernia Size - Length**	
N	92
Mean (SD)	11.33 (7.13)
**Hernia Size - Width**	
N	92
Mean (SD)	8.11 (4.84)
**Mesh Size at Implantation (cm^2^)**	
N	92
Mean (SD)	277.90 (112.76)
**Mesh Size - Length**	
N	92
Mean (SD)	17.22 (4.43)
**Mesh Size - Width**	
N	92
Mean (SD)	15.72 (4.65)
**Plane of Placement, n (%)**	
Retrorectus/TAR	48 (52.75%)
Intraperitoneal	42 (46.15%)
Retrofascial/Pre-Peritoneal	1 (1.10%)
Onlay	1 (1.10%)
**Primary Closure, n (%)**	
No	7 (7.61%)
Yes	85 (92.39%)
**Component Separation, n (%)**	
No	45 (48.91%)
Yes	47 (51.09%)
**If Component Separation = Yes, n (%)**	
Division of external oblique muscle anteriorly	22 (46.81%)
Division of transversus abdominis muscle posteriorly	25 (53.19%)
**Time in Surgery (Hours)**	
N	92
Mean (SD)	2.67 (1.34)

a Restricted to subjects with a Prior surgery.

Patients were considered at high risk for complications if they presented with one or more of the following comorbidities: BMI between 30 and 40, active smoker, COPD, diabetes mellitus, coronary artery disease, advanced age ( ≥ 75 years) [20,21]. BRAVO patients displayed obesity (55.43%), advanced age (11.96%), diabetes mellitus (21.74%), coronary artery disease (7.61%), active smoking (7.61%), and chronic obstructive pulmonary disease (COPD) (3.26%). The majority (78.26%) had at least one risk factor that could impact their recovery and put them at high risk of developing an SSO.

### 3.2 Intra-operative variables

The mean hernia defect was 112.05 ± 127.73 cm^2^ (Table 1) and the mean OviTex size was 277.90 ± 112.76 cm^2^. Repairs were completed primarily by open procedures (65.22%) as opposed to minimally invasive laparoscopic or robotic surgical procedures (34.78%) (Table 1). Component separation was performed in 51.09% of patients (Table 1). Most surgeons chose to place the implant either in the retrorectus/TAR (52.75%) or intraperitoneal (46.15%) plane, with only 1 patient (1.10%) having an implant placed either in the retrofascial/preperitoneal or onlay plane. Primary closure was achieved in 92.39% of patients (Table 1). On average, patients spent 2.67 ± 1.34 hours in surgery and remained in the hospital for 3.82 ± 2.95 days (Table 1). Chi-square and Kruskal-Wallis analyses did not determine any significant differences in intra-operative variables between subjects with and without 24-month follow-up.

### 3.3 Patient reported outcome assessments

**Fig. 2:**
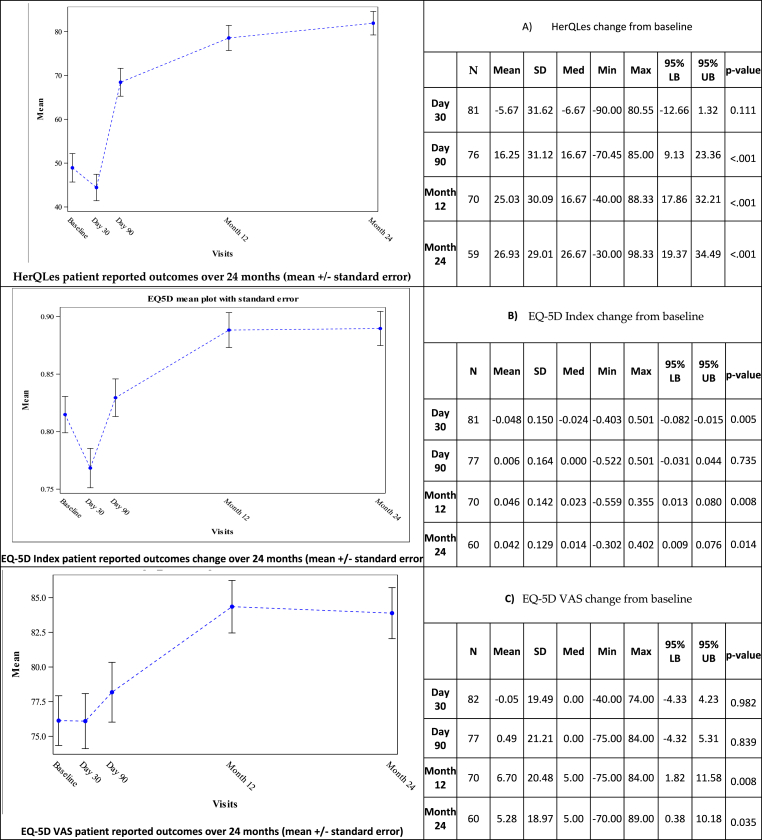
Patient reported outcomes, HerQles, EQ-5D VAS, EQ-5D Index.

The HerQLes assessment showed significant improvement from baseline in abdominal wall function and ability to do important daily activities at 3, 12, and 24 months (Fig. 2 A). The EQ-5D VAS assessment showed a significant increase in patient assessment of their health compared to baseline by 12 and 24 months. (Fig. 2 B), while the overall EQ-5D index scores also showed statistical improvement from baseline at 12, and 24 months (Fig. 2C).

### 3.4 Safety outcomes, complications, and risk factor analyses

**Fig. 3:**
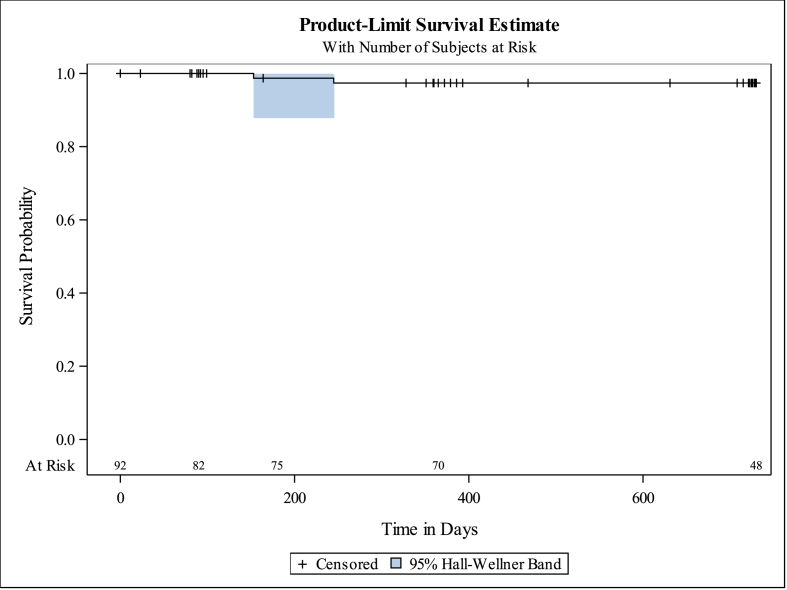
Kaplan-Meier curve for hernia recurrence over time (censored at day 730).

Unadjusted and time adjusted rates for hernia recurrence and adverse events to account for loss to follow up are presented in Table 4. The overall number of events, subjects, and unadjusted percentage of all subjects (N = 92) for events occurring up to 3 months post-surgery, as well as events occurring over the course of the entire study are also presented. Survival estimates are presented to account for patients not reaching the 3- and 24-month study timepoints. Subjects with follow-up beyond 730 days are censored at Day 730, corresponding to the exact 2-year anniversary. Of note, one of the recurrence events occurred after day 730 (relative day 796) and therefore was not included in the survival estimate. For completeness, that event was retained in the overall columns and accounted for in the overall hernia recurrence rate of 3.3% (row 1). The selection of censoring at Day 730 was used to provide stable estimates of the survival (i.e., the computed standard errors are small enough to provide valuable information). Had the study included longer-term follow-up rather than ending at two years, stable survival estimates may have been calculated out to 796 days. The risk of a patient experiencing a recurrence at the two-year time point was 2.6% based on the Kaplan Meier estimate (Fig. 3), while the overall study rate not accounting for lost to follow-up was 2.2% (2/92) at day 730 and 3.3% (3/92) at all timepoints as shown in Table 2. When considering only the patients who either returned for their 24-month follow-up visit (n = 65) or those that experienced a recurrence prior to 24 months and did not complete follow-up (n = 1), the unadjusted 24-month recurrence rate is conservatively 4.5% (3/66).

**Table 2:** Safety events by high- and low-level terms.

High level term	Low level term	Number Of Events	Subjects With Events	Overall % (n = 92)	Month 3% (n = 92)	Month 3 Adjusted %^a^	Month 24 Adjusted %^a^	95% LB	95% UB
**Hernia Recurrence**		3	3	3.3%	0.0%	0.0%	2.6%	0.7%	10.1%
Adjacent Recurrence		3	3	3.3%	0.0%	0.0%	2.6%	0.7%	10.1%
**SSO**		48	35	38.0%	34.8%	36.8%	40.7%	31.2%	52.0%
Seroma		12	12	13.0%	10.9%	11.6%	14.4%	8.4%	24.1%
Seroma (requiring intervention)		3	3	3.3%	3.3%	3.5%	3.5%	1.2%	10.5%
Hematoma		4	4	4.3%	4.3%	4.7%	4.7%	1.8%	12.0%
Wound Dehiscence		1	1	1.1%	1.1%	1.2%	1.2%	0.2%	8.1%
Skin Necrosis		1	1	1.1%	1.1%	1.2%	1.2%	0.2%	8.0%
Fistulae		2	2	2.2%	2.2%	2.3%	2.3%	0.6%	9.0%
Surgical site infection (SSI)		25	19	20.7%	16.3%	17.3%	23.3%	15.4%	34.3%
Superficial Infection		11	11	12.0%	9.8%	10.6%	14.0%	7.9%	24.2%
Deep/Abscess Infection		14	9	9.8%	6.5%	8.0%	10.6%	5.6%	19.4%
Organ Space Infection		0	0	0.0%	0.0%	–	–	–	–
**Complications**		11	11	12.0%	10.9%	11.5%	12.7%	7.3%	21.8%
Bowel Obstruction		1	1	1.1%	1.1%	1.2%	1.2%	0.2%	8.0%
Ileus		8	8	8.7%	8.7%	9.1%	9.1%	4.7%	17.4%
Any other non-surgery or Hernia-related complications		2	2	2.2%	1.1%	1.2%	2.5%	0.6%	9.6%
**Mesh Removal**		3	3	3.3%	2.2%	2.3%	3.6%	1.2%	10.7%
Full		2	2	2.2%	1.1%	1.1%	2.4%	0.6%	9.3%
Partial		1	1	1.1%	1.1%	1.2%	1.2%	0.2%	8.1%

a Kaplan Meier estimates censored at day 90 (3 months) 730 (24 months).

The most frequent SSO was surgical site infection (SSI) (20.7%), with superficial infection occurring in 12.0%, and deep/abscess infections occurring in 9.8% of patients (Table 2). Seroma formation occurred in 13% of patients, however, only 3.3% required intervention. Two patients (2.2%) had full matrix removals and one (1.1%) had a partial matrix removal (Table 4). One of the full matrix removals was due to a gastric ulcer perforation, where the revising surgeon proactively removed the matrix out of an abundance of caution even though the material appeared normal and intact. The second full removal occurred during the repair of an enterocutaneous fistula, which was deemed to be unrelated to the device, but where the takedown necessitated implant removal. In the patient requiring a partial mesh removal, a colon perforation led to infection at the site of OviTex implantation. During the removal it was noted that a portion of the material had become adhered to the small bowel and thus only a partial matrix removal was performed.

Risk factor analyses were performed on the incidence of recurrence and SSOs to determine the effect of any known patient risk factors on outcomes and safety events. Risk factor analysis of patients with hernia recurrences did not reveal any significant correlation, however, this was likely due to the low number of patients experiencing a recurrence. Similarly, risk factor analysis did not reveal any significant correlation between the risk of SSO and any comorbid condition.

## 4 Discussion

When treating a ventral hernia, surgeons must decide on an optimal surgical strategy to reduce the likelihood of recurrence and any potential downstream effects of adding foreign body reinforcement. Reinforced biologic matrices have been studied clinically, and have shown a low risk of serious SSOs, SSIs, complications, and recurrence rates in three ventral hernia studies [[22], [23], [24]] (Table 3). The utility of OviTex in ventral hernia repair is reinforced by both the previously published favorable results below and by the prospective 24-month BRAVO results recounted here.

**Table 3:** OviTex ventral hernia published studies.

	Parker, 2021 [22]		Ankney, 2021 [24]	Sivaraj, 2022 [23			
Total Pts	**50 OviTex**	50 Synthetic	259 **OviTex**	**36 OviTex**	51 NC-PADM^1^	17 C-PADM^2^	37 BADM^3^
Follow-Up	**12 Months**	12 Months	**1**–**5**9 **Months**	**28.6 median (±12.1)**	34.6 median (±15.2)	58.4 median (±19.4)	37.5 median (±17.5)
Patient Demographics	**CDC II+ (70%)**	CDC I (94%)	**--**	**CDC I-II (89%)**	CDC I-II (86.0%)	CDC I-II (94.2%)	CDC I-II (91.4%)
mVHWG	**II (32%)** **III (68%)**	II (94%) III (6%)	**--**	**I (33.4%)**	I (17%)	I (17.6%)	I (40.0%)
				**II (58.3%)**	II (78.7%)	II (70.6%)	II (51.4%%)
				**III (8.3%)**	III (4.3%)	III (11.8%)	III (8.6%)
Incidence of SSO	**36%**	22%	1.5**%**^b^	**16.7%**^a^	47.1%^a^	52.9%^a^	43.2%^a^
Incidence of SSI	**--**	–	0.8**%**^b^	**2.78%**	12.5%	11.8%	5.41%
Recurrence Rate	**6%**	12%	0.8**%**^b^	**2.78%**	13.7%	29.4%	24.3%

a Overall complications including SSI; 1 noncross-linked porcine acellular dermal matrix; 2 cross-linked porcine dermal biologic mesh; 3 fetal bovine acellular dermal matrix.

b SSO rate calculated (4/259), SSI rate calculated (2/259), recurrence rate calculated (2/259)

**Table 4:** Summary of recently published ventral hernia studies.

	DeNoto, 2022 (BRAVO)	Roth, 2021 (Phasix)[20]	Hope, 2022 (ATLAS)[21]	Harris, 2021 (PRICE)[25]	
Total Pts	92	121	120	82	83
Mesh Type	Reinforced tissue matrix	Resorbable Synthetic	Resorbable Synthetic	Biologic	Permanent Synthetic
Mesh Composition	Ovine forestomach matrix and polypropylene	Poly-4-hydroxybutyrate (P4HB)	Poly-4hydroxybutyrate (P4HB) with a hydrogel barrier	Porcine dermis	Polypropylene with hydrogel barrier or polypropylene
Trade Name	OviTex™ 1S Permanent^1^	Phasix™ Mesh^2^	Phasix-ST^TM^ Mesh^2^	Strattice^3^	Ventralight ST or Soft Mesh^4^
Follow-Up	24 Months	36 Months	24 Months	26 Months	
Surgical Approach	Open (65%) Laparoscopic (13%) Robotic (22%)	Open	Laparoscopic (55.8%) Robotic (44.2%)	Open	
Plane of Placement	Retrorectus (53%) Intraperitoneal (46%) Retrofascial/Pre-Peritoneal (1.1%) Onlay (1.1%)	Retrorectus (73%) Onlay (26%)	Intrabdominal	Onlay (17%) Sublay/Retrorectus (38%) Underlay (30%)	Onlay (28%) Sublay/Retrorectus (30%) Underlay (31%)
Primary Closure	92%	94%	N/A	85%	89%
Component Separation	51%	43.8%	1.7%	35%	46%
BMI (kg/m^2^)	31.0 ± 4.51	32.2 ± 4.5	33.2 ± 4.5	36.1 ± 9.6	35.0 ± 7.7
CDC Wound Class	I (80%) II (15%) III (4%)	I (100%)	I (100%)	I (67%) II (23%) III (1%) IV (9%)	I (70%) II (23%) III (0%) IV (7%)
Incidence of SSO/Complications	38% SSO(including SSI)	6.6% (Seroma req. int only) 15.7% (Device related AEs)	0.8%^a^<45 Days 0.0%^a^>45 Days (req. intervention0	21% SSO (excluding SSI)	22% SSO (excluding SSI)
Incidence of SSI	20.7%	9.3 ± 0.03%^a^	0%	39%	34%
Recurrence Rate (Final Follow Up Population)	4.5% (3/66)	–	–	40% (25/63)	22% (14/64)
Recurrence Rate(Kaplan Meier estimate)	2.6%^a^	17.9 ± 0.4%^a^	31.7% (18.6% defects <7.1 cm^2^ and 43.3% defects >7.1 cm^2^)^a^	–	–

a Kaplan Meier estimate 1: TELA Bio, Inc., Malvern, PA; 2: C.R. Bard, Inc., Warwick, RI; 3: LifeCell Corporation, Branchburg, NJ; 4: Becton, Dickinson & Company (BD), Franklin Lakes, NJ.

In the current study, there were no differences in baseline demographics or surgical characteristics of patients who completed 24-month follow up and those who did not, except for a significant difference in age. Patients who completed 24-month follow up were older than those that did not, possibly attributed to common reasons that younger patients don't return for an office visit, including scheduling conflicts with work or home, or that they are doing well clinically. Given that age is hypothesized to be associated with worse outcomes, the potential bias of having older patients in the 24-month cohort may be conservative. However, the BRAVO study was not designed to evaluate the impact of age on outcomes, therefore this observation would need to be investigated in future studies to determine the true cause and impact on clinical results.

BRAVO study recurrence rates and complications were analyzed using both unadjusted and time adjusted rates. The Kaplan-Meier estimate for recurrence rate at 24 months (day 730) was 2.6%, while the unadjusted overall recurrence rate was 4.5% (3/66) based on patients returning for their 2-year visit, or experiencing a recurrence prior to 24 months.

While risk factor analysis did not detect any significant predictors of recurrence, several variables were informally explored to identify potential factors in common for the three patients experiencing a recurrence. There was no commonality between mVHWG and CDC wound class as one mVHWG Grade I/CDC Class I, one mVHWG Grade II/CDC Class I and one mVHWG Grade III/CDC Class II patient experienced a recurrence. There was no commonality in surgical approach with recurrences observed in one open, one laparoscopic and one robotically treated patient. However, 3/3 patients experiencing a recurrence had underlay/sublay intraperitoneal placement of matrix and primary closure was not achieved in 1/3 of these patients. A larger study would be required to elucidate if plane of placement or primary closure is a definitive contributor to recurrence, as the incidence of recurrence in this study was too low to draw conclusions.

The overall SSI rate in the BRAVO study was 20.7%, while the Kaplan-Meier estimated rate was 23.3% at 24 months. Risk of surgical site infection is influenced by both procedural and patient variables, including patient comorbidities. Procedural factors include surgical technique, approach (open vs laparoscopic), concomitant procedures, and prolonged operative time. Of the patients experiencing an infection, 89.5% were classified as Grade II and III according to the mVHWG putting them at a higher risk for infection. The average procedure time was 2 h 58 min and the majority of patients (17/19, 90%) had open procedures. Seven (7/19, 36.8%) had a stoma, a concomitant procedure and/or a prior wound infection from a prior ventral hernia repair. Twelve (12/19, 63.2%) had a prior ventral hernia repair and the majority underwent component separation (13/19, 68.4%). While these patient and surgical characteristics may help to explain the observed SSI rate, further investigation is required to understand the discrepancy in the BRAVO SSI rate versus those previously published on this material.

The 24-month results of the BRAVO trial also demonstrate that the use of a reinforced biologic in ventral hernia repair improved patient reported quality of life. HerQLes scores significantly increased beginning as early as 3 months (90 days) (16.25 points) post-surgery and continuing at the 12- (25.03 points) and 24-month (26.93 points) timepoints (Fig. 2 A), exceeding the Minimal Clinically Important Difference (MCID) of 15.6 points established for the HerQLes assessment tool [26]. There was also a significant increase from baseline in EQ-5D VAS (Fig. 2 B) and EQ-5D index (Fig. 2C) scores at 12 and 24 months. Use of OviTex 1S therefore resulted in meaningful improvements in patient quality of life and perceived health as early as 90 days after surgery, and this improvement persisted over 24-months of follow-up.

Although not identical, the study design and patient cohort of the BRAVO study most closely aligns with the P4HB Phasix study by Roth et al. in open ventral hernia repair, the P4HB Phasix-ST study by Hope et al. in minimally invasive ventral hernia repair, and the Harris et al. study of Strattice and Ventralight ST or Soft Mesh in open ventral hernia repair [20,21,25]. These study results are summarized here in an effort to help contextualize the results of the current study (Table 4).

The Roth et al. Phasix study was a prospective, multi-center trial for primary ventral, primary incisional, or multiply-recurrent hernia in subjects at risk for complications with both 18- and 36-month published endpoints. One hundred twenty-one (121) patients were enrolled with 95 (79%) completing 18-month follow-up and 82 (67.8%) completing 36-month follow-up. The Kaplan Meier estimated hernia recurrence rate for the Roth et al. study was 9% at 18 months and 17.9% at 36 months. The Kaplan-Meier estimated overall SSI rate was 9.3%.

The Hope et al. ATLAS study was a prospective, multicenter 24-month trial evaluating P4HB-ST (Phasix ST) for laparoscopic or robotic ventral hernia repair in patients at high risk for SSOs. One hundred twenty (120) patients were treated, and 83 (69.2%) completed 24-month follow-up. The incidence of SSO was relatively low, possibly due to the population only undergoing minimally invasive surgery which typically has lower overall complications. Despite the low SSO rate, the Kaplan-Meier estimated hernia recurrence rate for the ATLAS study was 31.7% at 24-months (Day 730). Even when examining the results which were stratified for defect size, the recurrence rate for small defects (<7.1 cm^2^) treated with P4HB-ST was 18.7%.

The Harris et al. PRICE study was a single-blind, randomized controlled trial designed to evaluate whether an unreinforced porcine biologic (Strattice) or permanent synthetic (Ventralight ST or Soft Mesh) yielded lower recurrence and complication rates over a 24-month period after open ventral hernia repair. One hundred sixty-five (165) patients were enrolled, and 137 (83%) completed their 12-month follow up while 94 (57%) completed their 2-year follow-up. The recurrence rates reported in the PRICE study were calculated based on the number of subjects returning for their 2-year visit in each group, plus any recurrences which occurred prior to the 2-year time point. The overall recurrence rate for unreinforced biologics was 40% (25/63) at 2 years, while the overall recurrence rate was 22% (14/64) for permanent synthetic at the same time point. When looking at only Class I patients, the recurrence rates at 2 years were 34% (14/41) for unreinforced biologics and 28% (13/47) for permanent synthetics. It should be noted, however, that the PRICE study used imaging to diagnose hernia recurrence not discernible upon physical examination while the BRAVO study did not. The number of re-herniations only diagnosed through imaging was not reported in the PRICE study, however, recurrences are expected to be higher using imaging modalities as opposed to clinical exam only. The incidence of SSI was 39% and 34%, respectively, for patients treated with unreinforced biologic and permanent synthetic in the PRICE study. The inclusion of Class IV wounds in the PRICE trial (9% of unreinforced biologic patients and 7% of permanent synthetic patients) as well as the entire population undergoing open repair could have contributed to the relatively higher SSI rate.

### 4.1 Limitations

This is a non-randomized observational study with no comparisons to a direct control. Addition of a control arm could aid in definitively determining any direct effects of ventral hernia repair with a reinforced biologic. This study did not require a single surgical technique or plane of placement which may contribute to varying results. Assessment of recurrence was based primarily on clinical exam, potentially missing asymptomatic recurrences. Due to the heterogeneity of ventral hernia repair patients and the unique study design for each clinical trial, comparisons with results published in the literature should be made with caution. Long term follow-up visits occurred during the period of March 2020 through August 2021 when COVID 19 was having significant impacts on healthcare facilities and staff. To account for higher than expected lost to follow up, Kaplan Meier analysis was employed and these results are displayed alongside unadjusted observed results.

## 5 Conclusions

The final analysis of the BRAVO study provides evidence that ventral hernia repair with OviTex 1S results in a low rate of recurrence over a 24-month period. This is well past the expected timeframe for when the ovine matrix has been remodeled with host tissue and is no longer contributing to the strength of the repair. The differentiating features of a biologic matrix sourced from ovine forestomach which is reinforced with polymer may help explain the low hernia recurrence rates with OviTex 1S.

While the overall complication rate in this study was acceptable, surgical site infection was higher than expected. Several patient and surgical factors were identified that could have contributed to this SSI rate, however**,** the use of OviTex 1S must be studied more deliberately in future prospective trials to better understand this observation. Despite these unexpected results, patients treated with OviTex 1S experienced statistically significant and clinically meaningful improvements in their self-reported quality of life beginning at early time points which were sustained over a 24-month period.

Overall the BRAVO study demonstrates that use of the ovine reinforced tissue matrix OviTex 1S is a viable option for use in ventral hernia repair. Additional studies with longer term follow-up are needed to draw definitive conclusions on the use of OviTex 1S.

## Data availability statement

The datasets generated during and/or analyzed during the current study are available from the corresponding author on reasonable request.

## Provenance and peer review

Not commissioned, externally peer-reviewed.

## Ethical approval

All procedures performed in studies involving human participants were in accordance with the ethical standards of the institutional and/or national research committee and with the 1964 Helsinki declaration and its later amendments or comparable ethical standards. The protocol was approved by each center's Institutional Review Board or under the central approval (WIRB Pr. No.: 20142056).

## Sources of funding

Source of funding was TELA Bio, Inc.

## Author contribution

Conceptualization, G.D.III; methodology, G.D.III; formal analysis, G.D.III; investigation, G.D.III, E.P.C., S.J.P., M.S., G.S., M.T., G.T. and J.Y.; writing—review and editing, G.D.III, E.P.C., S.JP., M.S., G.S., M.T., G.T. and J.Y. All authors have read and agreed to the published version of the manuscript.

## Registration of research studies

1. Name of the registry: Clinicaltrials.gov
2. Unique Identifying number or registration ID: NCT03074474
3. Hyperlink to your specific registration (must be publicly accessible and will be checked): https://clinicaltrials.gov/ct2/show/NCT03074474

## Guarantor

George DeNoto.

## Consent

Informed consent was obtained from all individual participants included in the study. Written informed consent has been obtained from the patient to publish this paper.

## Declaration of competing interest

The funders had a role in the design of the study; in the collection, analyses, and interpretation of data; in the writing of the manuscript, and in the decision to publish the results.

George DeNoto, Salvatore Pacella, Michael Sawyer, and Geoffrey Slayden are all consultants for TELA Bio, Inc.
